# NiCo_2_O_4_ nanoparticles rich in oxygen vacancies: Salt-Assisted preparation and boosted water splitting

**DOI:** 10.3389/fchem.2022.996084

**Published:** 2022-09-15

**Authors:** Xiaobo He, Yuanchu Dong, Fengxiang Yin, Guoru Li, Xinran Zhao

**Affiliations:** ^1^ Jiangsu Key Laboratory of Advanced Catalytic Materials and Technology, School of Petrochemical Engineering, Changzhou University, Changzhou, China; ^2^ College of Chemical Engineering, Beijing University of Chemical Technology, Beijing, China

**Keywords:** salt-assisted method, spinel, hydrogen evolution reaction, oxygen evolution reaction, water splitting

## Abstract

NiCo_2_O_4_ is a promising catalyst toward water splitting to hydrogen. However, low conductivity and limited active sites on the surfaces hinder the practical applications of NiCo_2_O_4_ in water splitting. Herein, small sized NiCo_2_O_4_ nanoparticles rich in oxygen vacancies were prepared by a simple salt-assisted method. Under the assistance of KCl, the formed NiCo_2_O_4_ nanoparticles have abundant oxygen vacancies, which can increase surface active sites and improve charge transfer efficiency. In addition, KCl can effectively limit the growth of NiCo_2_O_4_, and thus reduces its size. In comparison with NiCo_2_O_4_ without the assistance of KCl, both the richer oxygen vacancies and the reduced nanoparticle sizes are favorable for the optimal NiCo_2_O_4_-2KCl to expose more active sites and increase electrochemical active surface area. As a result, it needs only the overpotentials of 129 and 304 mV to drive hydrogen and oxygen evolution at 10 mA cm^−2^ in 1 M KOH, respectively. When NiCo_2_O_4_-2KCl is applied in a symmetrical water splitting cell, a voltage of ∼1.66 V is only required to achieve the current density of 10 mA cm^−2^. This work shows that the salt-assisted method is an efficient method of developing highly active catalysts toward water splitting to hydrogen.

## 1 Introduction

Hydrogen is not only an important raw material, but also a fuel with high energy density (Tenhumberg and Büker, 2020; Hjeij et al., 2022; Lee et al., 2022). However, most of commercial hydrogen production technologies are accompanied by massive carbon dioxide emissions (Dawood et al., 2020). Among the recently-developed green methods to hydrogen, electrochemical water splitting in alkaline conditions has attracted much attention (Wang et al., 2019; Cao et al., 2020; Yu et al., 2021), due to its zero emission, high purity of the produced hydrogen and abundant sources. Electrochemical water splitting contains two half reactions: cathodic hydrogen evolution reaction (HER) and anodic oxygen evolution reaction (OER) (Zhu et al., 2020a). Both HER and OER involve the transfer of multiple electrons and protons, thus resulting in slow kinetics. Highly active HER and OER catalysts are indispensable to promote water splitting to hydrogen fast and efficiently, especially bifunctional catalysts that can simultaneously accelerate HER and OER. Till now, Pt-based and Ir-based catalysts are the state-of-the-art HER and OER electrocatalysts, respectively (Zhang et al., 2022a; Kim et al., 2022). However, they are limited in large-scale production due to high costs and low reserves. In recent years, various Co-based electrocatalysts, such as phosphides (Zhang et al., 2022b), carbides (Wang et al., 2021), oxides (Jung et al., 2021) and sulfides (Dong et al., 2022) have been developed and applied for water splitting to hydrogen. Among them, NiCo_2_O_4_ spinel oxide has shown great application potentials in many energy storage and conversion systems (Chen et al., 2018; Ranjani et al., 2018), because of simple preparation methods, high stability against corrosion in electrochemical systems, and, more importantly, high electrochemical activity (Ha et al., 2019; Du et al., 2021; Sun et al., 2021). And also, it has been applied as highly efficient electrocatalysts in water splitting to hydrogen. For instance, He et al. developed NiCo_2_O_4_@FeP_x_ core-shell nanoneedle arrays grown on nickel foam (FeP-NCO@NF) as highly active bifunctional catalysts toward both HER and OER. The synergetic effects between NiCo_2_O_4_ and FeP_x_ resulted in the low HER (∼82 mV) and OER (∼220 mV) overpotentials and the low overall water-splitting voltage (∼1.523 V) to deliver a current density of 10 mA cm^−2^ (He et al., 2021). Du et al. used *in-situ* deposition to control the loading of NiCo_2_O_4_ on the surface of Co_9_S_8_ by adjusting the number of deposition cycles (Du et al., 2021). The different loading of NiCo_2_O_4_ showed the different dominant activity toward HER and OER, respective. That is, Co_9_S_8_@NiCo_2_O_4_-70 cycles had the optimal OER activity, while Co_9_S_8_@NiCo_2_O_4_-10 cycles afforded the optimal HER activity. When they were coupled for overall water-splitting, Co_9_S_8_@NiCo_2_O_4_-70ǁCo_9_S_8_@NiCo_2_O_4_-10 displays a low voltage of ∼1.55 V to drive the cell at 10 mA cm^−2^. Further results demonstrated that NiCo_2_O_4_ promoted the adsorption and dissociation of water molecules in alkaline electrolytes.

Herein, this work has developed a salt (KCl)-assisted method to prepare NiCo_2_O_4_ nanoparticles as highly efficient catalysts toward HER, OER and overall water splitting. Recently, salt-assisted methods have been used to develop highly active catalysts towards electrocatalytic reactions (Li et al., 2020; Li et al., 2021a; Peng et al., 2021). Generally, salt-assisted methods have the two following main advantages. On one hand, the used salts can act as templates for the formation of porous (Li et al., 2021a) or specific nanostructures (Li et al., 2020). As a result, high specific surface areas have been achieved and the growth of nanostructures of catalysts can be tuned. On the other hand, it is favorable for salt-assisted methods to create abundant defects in catalysts (Peng et al., 2021), thus promoting the electrocatalytic processes.

Herein, the used KCl play important roles in tuning the structures of the resultant NiCo_2_O_4_, like other salts used in reports above (Li et al., 2020; Li et al., 2021a; Peng et al., 2021). As compared with NiCo_2_O_4_ without the assistance of KCl, the smaller-sized nanoparticles in the optimal NiCo_2_O_4_-2KCl are favorable to achieve the higher BET SSAs and expose more active sites and thus increase electrochemically active surface areas. Meanwhile, the KCl promotes the formation of abundant O_v_ in NiCo_2_O_4_-2KCl as well as NiCo_2_O_4_-1KCl and NiCo_2_O_4_-3KCl, and the more abundant O_v_ can also effectively improve the electrochemically active surface areas and enhance charge transfer efficiency during HER and OER. As a result, NiCo_2_O_4_-2KCl affords the much higher HER/OER bifunctional activity than NiCo_2_O_4_ without the assistance of KCl in alkaline electrolyte. Meanwhile, a cell consisting of NiCo_2_O_4_-2KClǁNiCo_2_O_4_-2KCl has the comparable overall water splitting performance to a 20 wt% Pt/CǁIrO_2_ cell.

## 2 Experimental

### 2.1 Preparation of catalysts

**FIGURE 1:**
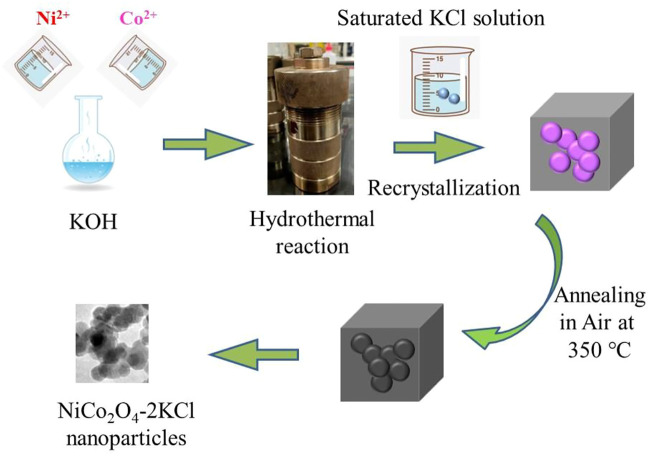
Schematic illustration of the salt-assisted preparation procedures of NiCo_2_O_4_.

Figure 1 shows the whole preparation procedures of NiCo_2_O_4_ nanoparticles via the salt-assisted method. To prepare the typical NiCo_2_O_4_-2KCl, 29.55 g (0.1 mol) of Co(NO_3_)_2_•6H_2_O, 59.34 g (0.2 mol) of Ni(NO_3_)_2_•6H_2_O were added into 320 ml of a aqueous solution of KOH (0.94 M) and stirred at room temperature for 1 h. Then, the suspension was transferred into a Teflon autoclave and heated at 200°C for 24 h. After naturally cooled to room temperature, the obtained NiCo precursor was washed by deionized water and absolute ethanol several times, and then was added into a saturated KCl solution that was prepared at 90°C by using 0.6 mol of KCl in advance. After stirring at 90°C for 30 min, a homogeneous suspension was formed, and then cooled to room temperature, filtered and dried at 60°C for the subsequent procedures. The obtained mixture consisting of NiCo precursor and KCl was annealed at 350°C in air for 2 h at a heating rate of 5°C min^−1^. The obtained black solid powder was washed with deionized water and absolute ethanol for several times. After dried at 60°C, NiCo_2_O_4_ nanoparticles rich in oxygen vacancies was achieved. Meanwhile, NiCo_2_O_4_ without the assistance of KCl was prepared as a comparison. In addition, other NiCo_2_O_4_-*n*KCl (*n* = 1, 1.5, 3) samples were also synthesized via the similar salt-assisted procedures, where *n* represents the molar ratio of the fed amount of KCl and the total fed amount of Co and Ni (0.3 mol).

### 2.2 Characterizations

X-ray powder diffraction (XRD) patterns were recorded on a diffractometer (D8 Advance, Bruker, Germany) with Cu K_α_ radiation (Cu K_α,_ λ = 1.5406 Å). X-ray photoelectron spectra (XPS) were collected on an ESCALAB 250XI XPS photoelectron spectrometer with an Al K_α_ X-ray resource (Thermo Fisher Scientific, United States) at a pass energy of 30 eV. The binding energy was referenced to the C 1s peak at 284.6 eV. Transmission electron microscopy (TEM) image was obtained on a microscope (200 kV, Tecnai G2 F20, FEI, United States). Electron paramagnetic resonance (EPR) spectra were acquired on a spectrometer (E500, Bruker, United States) under the X-band microwave (9.433 GHz, 0.998 mW) at 298 K with modulation frequency of 100 kHz, in which 2,2-Diphenyl-1-picrylhydrazyl (DPPH) was used as standard sample to analyze spin concentration. The N_2_ adsorption-desorption isotherms at 77 K were investigated on an analyzer (3Flex, Micromeritics, United States). The specific surface areas (SSAs) were analyzed via the Brunauer–Emmett–Teller (BET) method. Inductively coupled plasma-optical emission spectrometry (ICP-OES, Agilent ICP-OES 720, United States) was performed to determine the presence of Cl residues.

### 2.3 Electrochemical measurements

A three-electrode configuration and CHI760E electrochemical workstation were used to evaluate HER and OER activity in 1 M KOH (pH ≈ 14). The three electrodes include Ag/AgCl electrode with saturated KCl as reference electrode, carbon rod as counter electrode, and glassy carbon electrode (GCE) as working electrode that is connected to a rotating disk electrode (RDE) apparatus. Before preparing a GCE, a homogeneous dispersion of a sample including NiCo_2_O_4_-*n*KCl, 20wt% Pt/C or IrO_2_, was first prepared as follows: 2.5 mg of a sample, 2.5 mg of carbon black and 50 μL of Nafion solution were added into 1 ml of absolute ethanol, and the mixture was treated by sonication for 30 min. Then, ∼12.7 μL of the above dispersion was casted on the GCE with a diameter of 4 mm. After natural dried overnight, the GCE with a loading of ∼0.24 mg cm^−2^ was obtained. According to the Nernst equation *E* (vs. RHE) = *E* (vs. Ag/AgCl) + 0.197 + 0.0591 × pH, the actual electrode potential relative to Ag/AgCl is converted to that relative to RHE.

Before the test, cyclic voltammetry (CV) was performed at a scanning rate of 50 mV s^−1^ until the curve is stable. The linear sweep voltammetry (LSV) at a scan rate of 5 mV s^−1^ was used to record the polarization curve of a sample. The durability of HER (OER) activity was studied by the chronopotentiometry of 30000 s at a constant HER (OER) current density of 10 mA cm^−2^.

The electrochemically active surface area (EASA) is proportional to the electric double layer capacitance (*C*_DL_) of an electrocatalyst. Therefore, the *C*_DL_ can be used to indirectly indicate the electrochemical active area of a catalyst. First, the CV curve was collected with a sweep rate of 10–50 mV s^−1^ within the potential window without Faradic currents. Subsequently, the difference between cathode and anode current density (*j* = *j*_c_-*j*_a_) was plotted as a function of sweep speeds (ν), and the *C*_DL_ is the half of the slope of this linear plot. Electrochemical impedance spectroscopy (EIS) is obtained at an AC frequency of 10^6^–10^−1^ Hz under an open circuit voltage.

NiCo_2_O_4_-2KCl with the optimal bifunctional HER/OER activity was used as both cathode and anode catalysts in a symmetrical cell to test the overall water splitting performance, i.e., NiCo_2_O_4_-2KCl (cathode) ||NiCo_2_O_4_-2KCl (anode). Hydrophobic carbon papers (0.5 × 0.5 cm^2^) were used as working electrodes with the catalyst loading of ∼0.24 mg cm^−2^ in both electrodes. In 1 M KOH, a LSV is performed within the potential range of 1.4–1.9 V at 5 mV s^−1^. As a comparison, under the similar conditions, the performance of a water splitting cell with 20wt% Pt/C and IrO_2_ as the cathode and anode catalysts (20wt% Pt/C||IrO_2_), respectively, was also evaluated.

## 3 Results and discussion

### 3.1 Electrochemical catalytic performance

**FIGURE 2:**
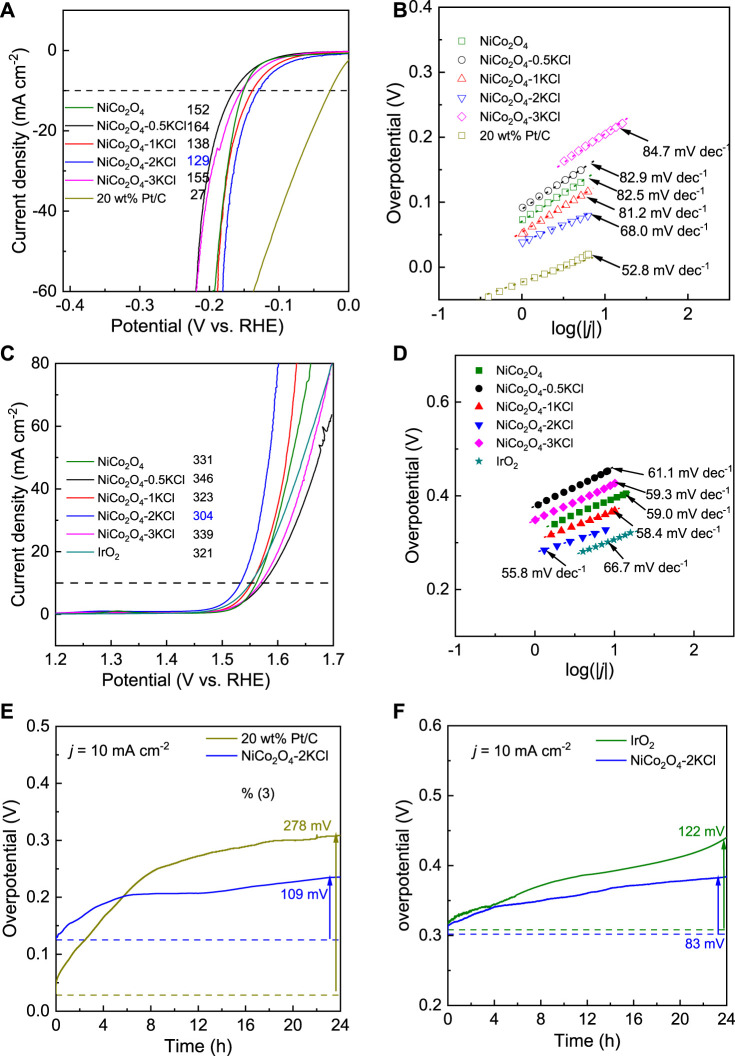
HER/OER activity and durability in 1 M KOH: **(A)** HER LSV curves; **(B)** HER Tafel plots; **(C)** OER LSV curves; **(D)** OER Tafel plots; **(E)** HER chronopotentiometry curves of 20 wt% Pt/C and NiCo_2_O_4_-2KCl at 10 mA cm^−2^; **(F)** OER chronopotentiometry curves of IrO_2_ and NiCo_2_O_4_-2KCl at 10 mA cm^−2^.

During the similar preparations, a series of NiCo_2_O_4_ samples were first prepared under the assistance of different salts, including KCl, KBr, KI, K_2_SO_4_ and KNO_3_, in order to screening the optimal salt. Supplementary Figures S1A,B show the corresponding HER and OER LSV curves in 1 M KOH. As shown in Supplementary Figure S1C, NiCo_2_O_4_-2KCl shows the lowest overpotentials at 10 mA cm^−2^ toward both HER and OER, which demonstrates that the assisting effects of KCl on promoting HER and OER activity are better than the other salts. Thus, the assisting effects of different fed amounts of KCl were further evaluated. Figure 2A shows the HER LSV curves of NiCo_2_O_4_-*n*KCl and NiCo_2_O_4_. NiCo_2_O_4_ (without the assistance of KCl) needs an overpotential (η_10-HER_) of ∼152 mV to reach 10 mA cm^−2^. However, the η_10-HER_ of NiCo_2_O_4_-0.5KCl is slightly increased to ∼164 mV relative to NiCo_2_O_4_. After further increasing the fed amount of KCl, the η_10-HER_ decreases and subsequently increases. In other word, NiCo_2_O_4_-2KCl has the smallest η_10-HER_ (∼129 mV), though the η_10-HER_ is still higher than that of 20wt% Pt/C (∼27 mV). Figure 2B exhibits the corresponding HER Tafel plots. The Tafel slopes show a change trend similar to that of η_10-HER_ values. Although the Tafel slope of NiCo_2_O_4_-2KCl (∼68 mV dec^−1^) is higher than that of 20 wt% Pt/C (∼52.8 mV dec^−1^), it is still the lowest one among the prepared NiCo_2_O_4_-*n*KCl samples, also lower that of NiCo_2_O_4_ (∼82.5 mV dec^−1^). It indicates the faster HER kinetic as compared with other samples, following Heyrovsky and Tafel reactions as rate-determining steps (Zhang et al., 2018a). In addition, the OER LSV curves are shown in Figure 2C. The change trend of the OER overpotential (η_10-OER_) along with the fed amounts of KCl is similar to that for η_10-HER_ (Figure 2A). Similar to the HER activity, NiCo_2_O_4_-2KCl has the lowest η_10-OER_ (∼304 mV) among the prepared samples, which is lower than that of IrO_2_ (∼321 mV). Figure 2D exhibits the corresponding OER Tafel plots. NiCo_2_O_4_-2KCl has the lowest Tafel slope of ∼55.8 mV dec^−1^, also lower than that of IrO_2_ (∼66.7 mV dec^−1^), indicating the highest OER kinetics among the prepared samples. The above results demonstrate that the fed amount of KCl significantly affects the HER and OER activity of NiCo_2_O_4_ samples, and a moderate amount of KCl is required to optimize both HER and OER activity. Table 1 shows the summary of HER and OER activity of some typical electrocatalysts reported in the literatures. Obviously, the HER and OER catalytic performance of NiCo_2_O_4_-2KCl is comparable to that of the recently-developed HER/OER bifunctional catalysts, although the highly conductive substrates were not applied to support as the binder-free electrodes, such as carbon cloth (Dong et al., 2022), Ni foams (Ha et al., 2019; Du et al., 2021; He et al., 2021), etc.

**TABLE 1:** The summary of HER and OER activity and overall water splitting performance of the recently reported catalysts in 1 M KOH.

Catalysts	Substrates	η_10−HER_ (mV)	η_10−OER_ (mV)	Overall water splitting voltage @ 10 mA cm^−2^ (V)	References
**NiCo**_**2**_ **O**_**4**_ **-2KCl**	**GCE (binder)** ^a^	**129**	**304**	**1.66**	**This work**
FeOOH@NiCo_2_O_4_	Ni foil (binder)	146	203	1.58	Cao et al. (2020)
Amorphous Ni-Co-S nanoflake arrays	Carbon cloth (binder-free)^b^	192	296	1.60	Dong et al. (2022)
Co_9_S_8_@NiCo_2_O_4_	Ni foam (binder-free)	104	270@100 mA cm^−2^	1.55	Du et al. (2021)
N-NiCo_2_O_4_@C	Ni foam (binder-free)	42	242	1.43	Ha et al. (2019)
FeP-NCO	Ni foam (binder-free)	82	220	1.523	He et al. (2021)
NiCo_2_O_4_ hollow microcuboids	Ni foam (binder)	110 in 1 M NaOH	290 in 1 M NaOH	1.65	Gao et al. (2016)
NiCo_2_O_4_/Cu_*x*_O	Cu foam (binder-free)	92	213	1.61	Ouyang et al. (2021)
Cobalt phosphide/N-doped carbon nanotubes	GCE (binder)	94	317	1.619	Yang et al. (2021)
CoP@FeCoP/N-doped carbon	Carbon paper (binder)	141	238	1.68	Shi et al. (2021)
NiCoP nanorod array	Ni foam (binder-free)	60	153	1.55	Hu et al. (2021)
Co-Mo_2_C@N-doped carbon	GCE (binder)	92	338	1.68	Zhang et al. (2021)

a “Binder” represents that the preparation of an electrode needs polymer binders to bind powder-like catalysts on the surface of the electrode.

b “Binder-free” means that the target catalyst has grown directly on the used substrate to form a self-supported electrode.

According to the above discussion, NiCo_2_O_4_-2KCl with the moderate amount of KCl (*n* = 2) assisted has the optimized HER and OER activity. Further, chronopotentiometry in 1 M KOH was used to evaluate the durability of HER and OER activity of NiCo_2_O_4_-2KCl plus noble metal benchmarked samples (i.e., 20wt% Pt/C as HER benchmarked catalyst and IrO_2_ as OER benchmarked catalyst). As shown in Figure 2E, the Δη_10-HER_ of 20 wt% Pt/C is high up to ∼278 mV after a running of 24 h, while the Δη_10-HER_ of NiCo_2_O_4_-2KCl is only ∼109 mV. As shown in Figure 2F, the Δη_10-OER_ of IrO_2_ and NiCo_2_O_4_-2KCl are ∼122 and ∼83 mV, respectively, when the running time of OER processes at 10 mA cm^−2^ is 24 h, showing the lower Δη_10-OER_ of NiCo_2_O_4_-2KCl. The evaluation results clearly shows that NiCo_2_O_4_-2KCl has the excellent HER and OER durability, and has the potential as a bifunctional overall water splitting catalyst.

**FIGURE 3:**
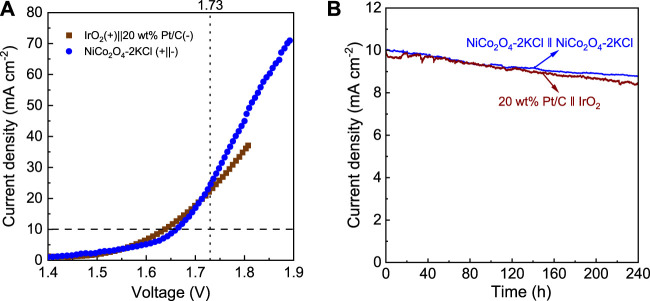
**(A)** LSV curves of overall water splitting in 1 M KOH for NiCo_2_O_4_-2KCl|| NiCo_2_O_4_-2KCl and 20 wt% Pt/C||IrO_2_ cells; **(B)** *I*-t curves of NiCo_2_O_4_-2KCl|| NiCo_2_O_4_-2KCl (at ∼1.66 V) and 20 wt% Pt/C||IrO_2_ cells (at ∼1.63 V) for 240 h.

In view of the excellent HER and OER activity and durability of NiCo_2_O_4_-2KCl, it was used as both cathode and anode catalyst for a water splitting cell. For comparison, 20 wt% Pt/C (cathode, HER)||IrO_2_ (anode, OER) cell was also used for water splitting. Figure 3A shows the LSV curves of water splitting of the 2 cells. 20 wt% Pt/C||IrO_2_ needs ∼1.63 V to drive a current density of 10 mA cm^−2^, while NiCo_2_O_4_-2KCl||NiCo_2_O_4_-2KCl requires a slightly higher voltage of ∼1.66 V to achieve 10 mA cm^−2^. In addition, when the voltage exceeds 1.73 V, the overall water splitting current density of NiCo_2_O_4_-2KCl||NiCo_2_O_4_-2KCl is higher than that of 20 wt% Pt/C||IrO_2_. It suggests that the overall water splitting performance of NiCo_2_O_4_-2KCl||NiCo_2_O_4_-2KCl is comparable to that of 20 wt% Pt/C||IrO_2_ and other cells (Table 1). Furthermore, as shown in Figure 3B, NiCo_2_O_4_-2KCl||NiCo_2_O_4_-2KCl can run stably for 240 h at ∼1.66 V with the higher current retention of ∼88% as compared with the 20 wt% Pt/C||IrO_2_ (∼84% at 1.63 V) that also runs for 240 h.

All in all, NiCo_2_O_4_-2KCl, which is prepared by the assistance of KCl with the moderate fed amounts, affords the optimal HER, OER and overall water splitting catalytic performance.

### 3.2 Structural features

**FIGURE 4:**
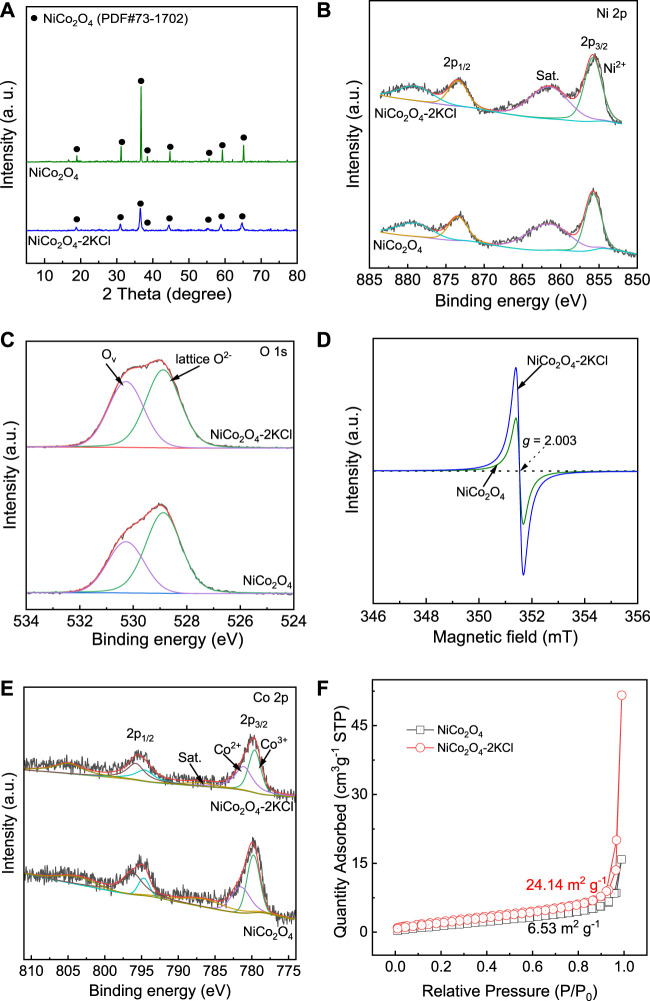
Structural characterizations of NiCo_2_O_4_ and NiCo_2_O_4_-2KCl: **(A)** XRD patterns; **(B)** XPS Ni 2p spectra; **(C)** XPS O 1s spectra; **(D)** EPR spectra; **(E)** XPS Co 2p spectra; **(F)** N_2_ adsorption-desorption isotherms at 77 K.

Figure 4A shows the XRD patterns of NiCo_2_O_4_ without the assistance of KCl and NiCo_2_O_4_-2KCl. All the diffraction peaks are consistent with the ones of PDF# 73-1702, which are all attributed to NiCo_2_O_4_ with inverse spinel structure. Generally, the narrower the full width at half maxima (FWHM) of diffraction peaks, the larger the grain size. According to the Scherer equation *D* = (K×γ)/(*B×*cos*θ*), in which *D* is the average grain size, K is the Scherrer constant, γ is wavelength of X-ray, *B* is FWHM of diffraction peaks, and *θ* is the half of Bragg angle, the average grain sizes of NiCo_2_O_4_ and NiCo_2_O_4_-2KCl can be estimated. Thus, the mean grain size of NiCo_2_O_4_ is larger (∼93 nm) and the crystallinity is high. By contrast, the diffraction peak intensities of NiCo_2_O_4_-2KCl are obviously weakened, and the FWHM is obviously broadened, which indicates that its crystallinity has decreased and the average grain size has become much smaller (∼35 nm). It suggests that the used KCl significantly limits the growth of NiCo_2_O_4_ nanoparticles.

Figure 4B shows the XPS Ni 2p spectra of NiCo_2_O_4_ and NiCo_2_O_4_-2KCl. For both NiCo_2_O_4_ and NiCo_2_O_4_-2KCl, only the peaks attributed to Ni^2+^ are deconvoluted and located at ∼855.8 eV (Bao et al., 2021), while the broad ones at ∼861.6 eV are ascribed to the satellite peaks, which suggests that the chemical states of Ni^2+^ are similar in them. As shown in Figure 4C, NiCo_2_O_4_-2KCl has more O_v_ and thus the higher O_v_/O^2-^ (lattice oxygen) molar ratio of ∼0.81 as compared with NiCo_2_O_4_ (∼0.59 of O_v_/O^2-^ molar ratio). The EPR spectra (Figures 4D, Supplementary Figure S2) with *g*-factor of ∼2.003 that is close to that of free electrons further confirm that all samples have more or less O_v_. The samples with the assistance of KCl except NiCo_2_O_4_-0.5KCl (∼1.67 × 10^14^ spins g^−1^) are generally richer in O_v_ with the higher spin concentration (∼7.68 × 10^14^ spins g^−1^ for NiCo_2_O_4_-2KCl, ∼5.67 × 10^14^ spins g^−1^ for NiCo_2_O_4_-1KCl, ∼4.84 × 10^14^ spins g^−1^ for NiCo_2_O_4_-3KCl) as compared with NiCo_2_O_4_ (∼3.89 × 10^14^ spins g^−1^) without the assistance of KCl, due to the ability of more O_v_ to capture more unpaired electrons (Kaftelen et al., 2012; Chen et al., 2021). Obviously, the more O_v_ formed in NiCo_2_O_4_-*n*KCl is closely related with the assistance of KCl during the preparations. To balance charge distribution, some parts of Co^3+^ at ∼779.7 eV are reduced to Co^2+^ (Yan et al., 2018), which is indicated by Co 2p spectra (Figure 4E). Although there is additional Co^2+^ in both NiCo_2_O_4_ and NiCo_2_O_4_-2KCl, on one hand, the binding energy of Co^2+^ in NiCo_2_O_4_-2KCl (∼781.1 eV) is lower than that in NiCo_2_O_4_ (∼781.5 eV); on the other hand, the Co^2+^/Co^3+^ molar ratio in NiCo_2_O_4_-2KCl (∼1.00) is higher than that in NiCo_2_O_4_ (∼0.72). The results of Co 2p spectra suggest that the relatively more Co^3+^ is reduced to Co^2+^ in NiCo_2_O_4_-2KCl as compared with NiCo_2_O_4_.

The N_2_ adsorption-desorption isotherms at 77 K of NiCo_2_O_4_-2KCl and NiCo_2_O_4_ are shown in Figure 4F. The corresponding BET SSA of NiCo_2_O_4_-2KCl is ∼24.14 m^2^ g^−1^, which is ∼3.7 times of that of NiCo_2_O_4_ (∼6.53 m^2^ g^−1^). Thus, KCl play an important role in achieving the higher SSA for NiCo_2_O_4_ during the preparations, which is consistent with the average grain sizes of them determined by Scherer equation.

**FIGURE 5:**
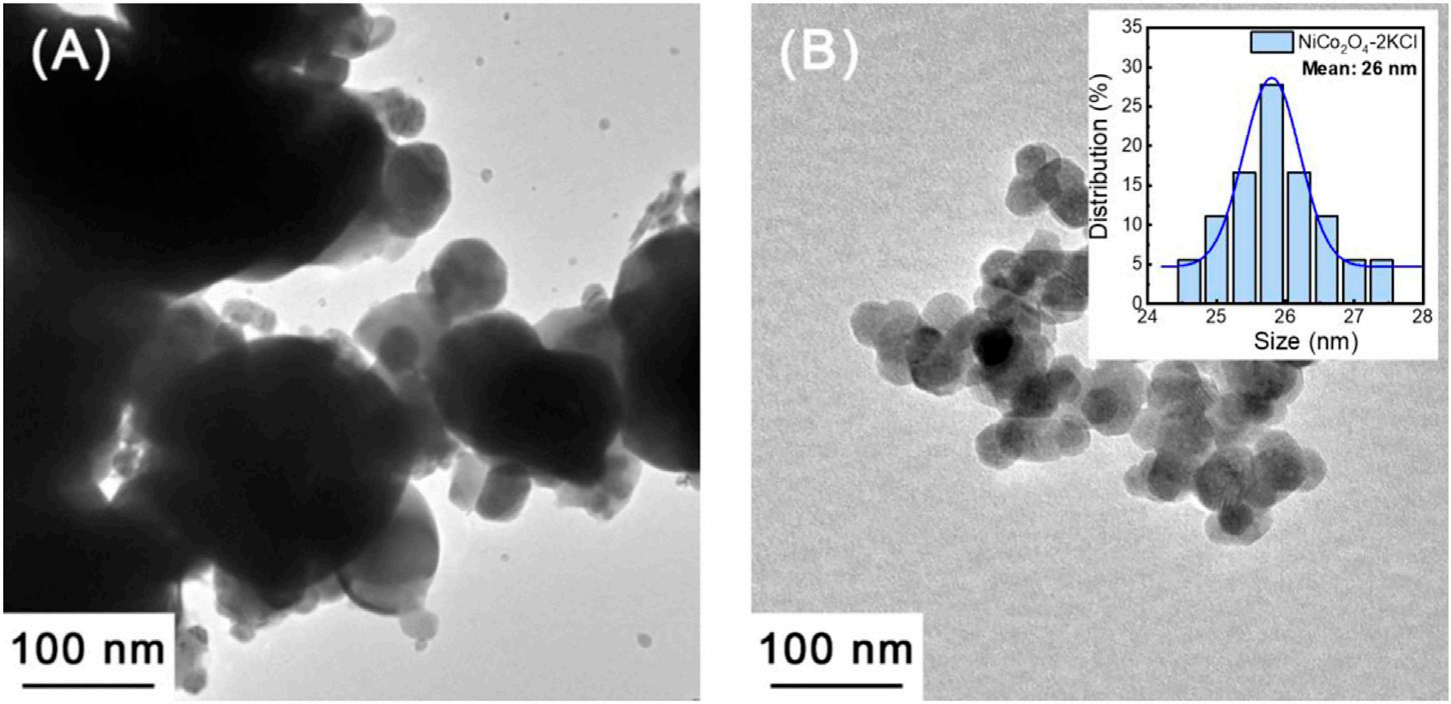
TEM images: **(A)** NiCo_2_O_4_ and **(B)** NiCo_2_O_4_-2KCl.

Figure 5A shows the TEM image of NiCo_2_O_4_ synthesized without the assistance of KCl. Obviously, the size distribution of NiCo_2_O_4_ particles is uneven. Quite large and very small particles coexist in this sample. The mean grain size of NiCo_2_O_4_ can be referred to the XRD results (∼93 nm). In comparison with NiCo_2_O_4_, NiCo_2_O_4_-2KCl has a smaller mean particle size (∼26 nm in inset of Figure 5B), and the size distribution is relatively narrower and more uniform. The results of TEM indicate that the KCl does effectively reduce the mean sizes of NiCo_2_O_4_ nanoparticles, which is consistent with the XRD results. In addition, as shown in Supplementary Figure S3, the unwashed intermediate after annealed contains KCl and NiCo_2_O_4_ nanoparticles, and KCl nanoparticles combine with NiCo_2_O_4_ nanoparticles tightly, thus playing the role in inhibiting the growth of NiCo_2_O_4_ nanoparticles.

### 3.3 Further brief discussion on catalytic performance

**FIGURE 6:**
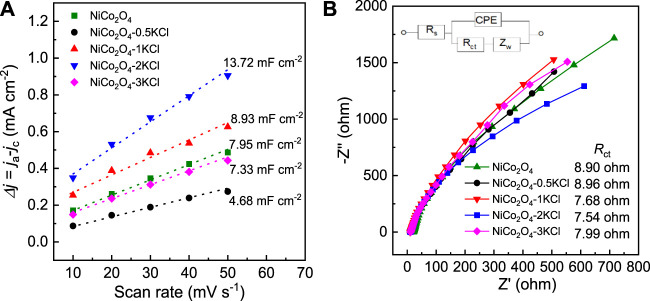
**(A)** The relationship between Δ*j* (= *j*_a_-*j*_c_) and scan rate; **(B)** EIS spectra at open circuit voltage, and the inset is the corresponding equivalent circuit.

The CV curves within the potential window without Faradic currents were used to determine *C*_DL_, because *C*_DL_ is proportional to the EASA of a catalyst (Gao et al., 2020; Wang et al., 2020). The corresponding CV curves are shown in Supplementary Figure S4, and the linear relationship between the difference of cathode and anode current density (*j* = *j*_c_-*j*_a_) and scan rate is shown in Figure 6A. With the increase of *n*, the *C*_DL_ values obtained from the half of the slopes of the linear plots show a trend similar to that for the HER or OER activity. Among NiCo_2_O_4_ and the NiCo_2_O_4_-*n*KCl samples, the *C*_DL_ of NiCo_2_O_4_-2KCl is largest, as high as ∼13.72 mF cm^−2^ Figure 6B shows the EIS spectra of NiCo_2_O_4_ and the NiCo_2_O_4_-*n*KCl samples. The charge transfer resistance (*R*_ct_) value of NiCo_2_O_4_-2KCl is lowered to 7.54 ohm, indicating the faster charge transfer between the GCE loaded with it and the electrolyte during the electrocatalysis as compared with the other samples.

As the ICP-OES results shown, there is no Cl residues detected within the detection limits of the used analyzer after three tests of every samples. Hence, KCl plays the indirect but important roles in electrocatalytic performance. According to the above results, KCl has great influences on the structures and surface compositions of NiCo_2_O_4_. The XRD and TEM results demonstrate that the average size of NiCo_2_O_4_-2KCl nanoparticles is significantly smaller than that of NiCo_2_O_4_. This indicates that KCl restricts the growth of NiCo_2_O_4_ nanoparticles. The smaller size of NiCo_2_O_4_-2KCl nanoparticles is favorable to exposing more active sites. In addition, the amount of O_v_ in NiCo_2_O_4_-2KCl is significantly increased as compared with NiCo_2_O_4_. Some studies have shown that oxides are more likely to form O_v_ under confinement effects (Kotomin et al., 2011; Arrigoni et al., 2016; Ye et al., 2022), and the generation energy of O_v_ in a small space becomes lower. Herein, KCl particles can limit the growth of NiCo_2_O_4_ nanoparticles due to the tight integration with each other (Supplementary Figure S3). When NiCo_2_O_4_ is gradually formed during annealing, a large number of O_v_ will be generated. Many studies have shown that O_v_ has been proven to exert favorable roles in promoting HER (Zhang et al., 2018b; Li et al., 2021b; Lu et al., 2021) and OER (Liu et al., 2019; Xiao et al., 2020; Zhong et al., 2021) activity in alkaline conditions, mainly because the O_v_ lowers the coordination numbers of active metal ions, and thus modifies their electronic structures and further tunes the adsorption strength of HER and OER intermediates (*H for HER, and *OH, *O and *OOH for OER) on them and the O_v_ also can decrease the energy barriers during HER or OER processes (Zhu et al., 2020b; Badreldin et al., 2021; Wu et al., 2021). Together plus the effects of the smaller sizes, NiCo_2_O_4_-2KCl with the more O_v_ can expose more active sites on its surface and achieves the higher EASAs (Figure 6A) as compared with NiCo_2_O_4_. In addition to favorable contributions to active sites, O_v_ can efficiently capture unpaired electrons and form additional energy levels in the band gap of NiCo_2_O_4_ samples, thus increasing the concentration of carriers and enhancing the charge transfer efficiency during electrochemical processes (Shin et al., 2020; Badreldin et al., 2021), which is indicated by the corresponding EIS spectra (Figure 6B) and the relationship between spin concentrations (proportional to the contents of O_v_) and *R*_ct_ values (Supplementary Figure S5). The higher charge transfer efficiency is more conducive to make full use of the exposed active sites and thus the activity of HER and OER. In summary, for NiCo_2_O_4_-2KCl, the small size and abundant O_v_ provide significant roles on promoting HER and OER with high bifunctional catalytic activity.

## 4 Conclusion

In this work, a salt-assisted method was used to prepare small-sized NiCo_2_O_4_ with abundant O_v_ as highly active catalyst for HER, OER and overall water splitting. KCl is used as a good agent to limit the growth of NiCo_2_O_4_ nanoparticles. The confinement effects resulted from KCl during the calcination processes effectively increases the amount of O_v_ in NiCo_2_O_4_ nanoparticles, thereby increasing the densities of active sites and enhancing charge transfer efficiency. As compared with NiCo_2_O_4_ without the assistance of KCl, both the reduced nanoparticle size and the more O_v_ are favorable for the optimal NiCo_2_O_4_-2KCl to expose more active sites and increase the EASA. As a result, the optimal NiCo_2_O_4_-2KCl exhibits the higher bifunctional catalytic activity of both HER and OER than NiCo_2_O_4_ and the other NiCo_2_O_4_-*n*KCl samples. When NiCo_2_O_4_-2KCl is used as both cathode and anode for overall water splitting, it can drive NiCo_2_O_4_-2KCl||NiCo_2_O_4_-2KCl cell to have the similar performance to that of 20 wt% Pt/C||IrO_2_ cell. This work provides an efficient method to prepare highly active catalyst toward electrocatalytic water splitting to hydrogen.

## Data Availability

The original contributions presented in the study are included in the article/Supplementary Material, further inquiries can be directed to the corresponding author.
