# Supplementary material for: NiCo2O4 nanoparticles rich in oxygen vacancies: Salt-Assisted preparation and boosted water splitting
Source: Front Chem. 2022 Sep 15;10:996084. doi: 10.3389/fchem.2022.996084 (PMC9520992; doi:10.3389/fchem.2022.996084)
Supplement: Supplementary file 1 [file DataSheet1.docx]

***Supporting Material***

**NiCo_2_O_4_ nanoparticles rich in oxygen vacancies: salt-assisted preparation and boosted water splitting**

Xiaobo He^1†^, Yuanchu Dong^1†^, Fengxiang Yin^1*^, Guoru Li^1^, Xinran Zhao^2^

^1^Jiangsu Key Laboratory of Advanced Catalytic Materials and Technology, School of Petrochemical Engineering, Changzhou University, Changzhou 213164, P. R. China

^2^College of Chemical Engineering, Beijing University of Chemical Technology, Beijing 100029, PR China

*** Corresponding author**

Tel.: +86-519-86330253

E-mail: yinfx@cczu.edu.cn (F. Yin)

† These authors contributed equally.

|  |  |
| --- | --- |
|  | |
| **Figure S1.** (A) HER and (B) OER LSV curves in 1 M KOH of NiCo_2_O_4_ samples prepared by the assistance of different salts; (C) Comparison of the HER and OER overpotentials for these samples. | |
|  | |

|  |
| --- |
| **Figure S2.** EPR spectra of NiCo_2_O_4_-0.5KCl, NiCo_2_O_4_-1KCl and NiCo_2_O_4_-3KCl. |

| 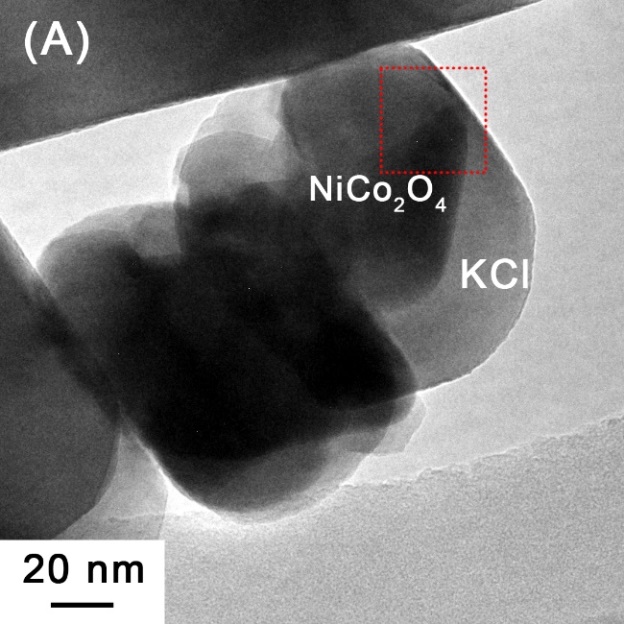 | 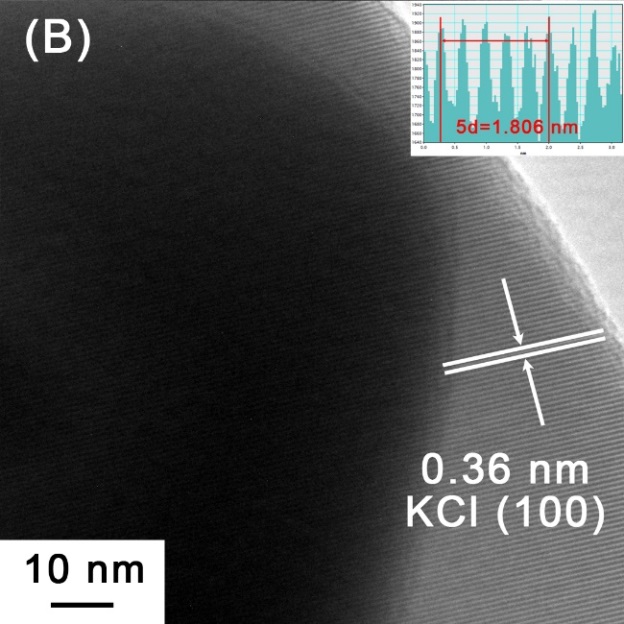 |
| --- | --- |
| 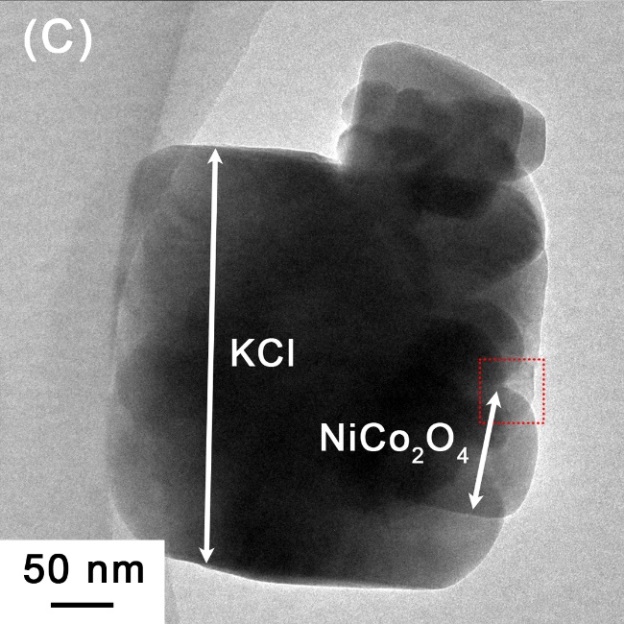 | 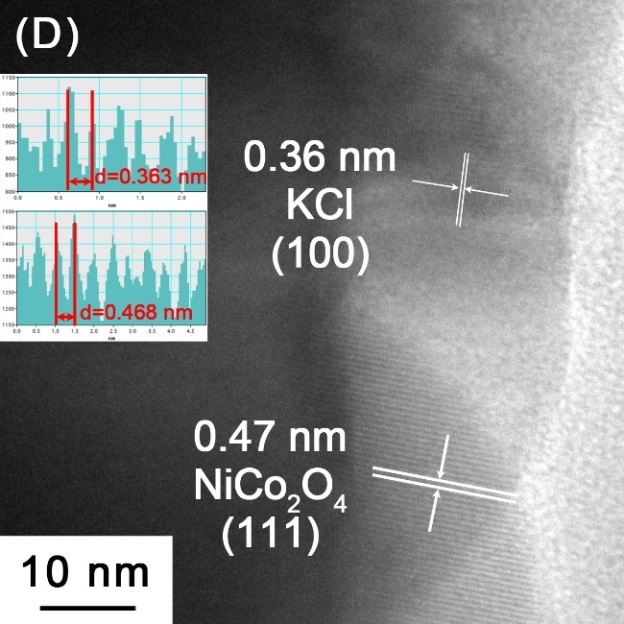 |
| **Figure S3.** (A-D) TEM images of the unwashed NiCo_2_O_4_-2KCl intermediate sample after annealing. | |

|  |  |
| --- | --- |
|  |  |
|  | |
| **Figure S4.** CV curves: (A) NiCo_2_O_4_ and (B)-(E) NiCo_2_O_4_-*n*KCl within 0.1 V windows around open-circuit potentials with different scanning rate in 1 M KOH. | |

|  |
| --- |
| **Figure S5.** Relationship between spin concentrations and *R*_ct_ values. |
